# Reframing the Technology Narrative: Subjective Aging and Inclusive Co-Design

**DOI:** 10.1093/geroni/igaf122.4183

**Published:** 2025-12-31

**Authors:** Pallabi Bhowmick, Erik Stolterman Bergqvist

**Affiliations:** University of Illinois Urbana-Champaign, Champaign, Illinois, United States; Indiana University Bloomington, Bloomington, Indiana, United States

## Abstract

Older adults’ technology acceptance is shaped not only by functional needs but also by subjective perceptions of aging and ageist stereotypes. While older adults acknowledge the value of assistive technologies, they often view these tools as more suitable for ‘others’, those older or in poorer health, rather than for themselves. This reluctance to see themselves as potential beneficiaries, coupled with stigma around needing assistance, poses barriers to adoption. For designing a technology to support social connection, we organized a co-design workshop with 15 older adults (ages 70–90, M = 78.1, SD = 6.4) and 6 student designers, deliberately structuring it to create a non-stigmatizing environment that fostered effective engagement. First, a humorous video of a Generation Z teenager struggling with a rotary phone reframed difficulties with new technologies as a universal experience. Second, a “technology timeline” activity highlighted older adults’ history of technological adaptability, countering stereotypes of technological ineptitude. Third, the “Someone Who Is Not Me” (SWIM) technique shifted the focus from personal need to a hypothetical ‘other’, reducing self-stereotyping while still enabling reflection on their own potential use. Interestingly, during ideation, participants transitioned from designing for imagined personas to actively situating themselves as end-users, expressing interest in integrating the technology into their lives. When deficit-based framings were avoided, older adults were receptive to adopting later-life technologies. This work underscores the importance of framing technology for older adults as supporting independence rather than signaling decline, and highlights how strategies that reduce stigma during co-design challenge stereotypes and empower older adults as producers of innovation.

